# Effects of Arbuscular Mycorrhizal Fungi on Rice Growth Under Different Flooding and Shading Regimes

**DOI:** 10.3389/fmicb.2021.756752

**Published:** 2021-10-26

**Authors:** Yutao Wang, Xiaozhe Bao, Shaoshan Li

**Affiliations:** Key Laboratory of Ecology and Environmental Science in Guangdong Higher Education and Guangdong Provincial Key Laboratory of Biotechnology for Plant Development, School of Life Sciences, South China Normal University, Guangzhou, China

**Keywords:** arbuscular mycorrhizal fungi, carbon-phosphorus exchange, defense, flooding, rice, shading

## Abstract

Arbuscular mycorrhizal fungi (AMF) are present in paddy fields, where they suffer from periodic soil flooding and sometimes shading stress, but their interaction with rice plants in these environments is not yet fully explained. Based on two greenhouse experiments, we examined rice-growth response to AMF under different flooding and/or shading regimes to survey the regulatory effects of flooding on the mycorrhizal responses of rice plants under different light conditions. AMF had positive or neutral effects on the growth and yields of both tested rice varieties under non-flooding conditions but suppressed them under all flooding and/or shading regimes, emphasizing the high importance of flooding and shading conditions in determining the mycorrhizal effects. Further analyses indicated that flooding and shading both reduced the AMF colonization and extraradical hyphal density (EHD), implying a possible reduction of carbon investment from rice to AMF. The expression profiles of mycorrhizal P pathway marker genes (*GintPT* and *OsPT11*) suggested the P delivery from AMF to rice roots under all flooding and shading conditions. Nevertheless, flooding and shading both decreased the mycorrhizal P benefit of rice plants, as indicated by the significant decrease of mycorrhizal P responses (MPRs), contributing to the negative mycorrhizal effects on rice production. The expression profiles of rice defense marker genes *OsPR1* and *OsPBZ1* suggested that regardless of mycorrhizal growth responses (MGRs), AMF colonization triggered the basal defense response, especially under shading conditions, implying the multifaceted functions of AMF symbiosis and their effects on rice performance. In conclusion, this study found that flooding and shading both modulated the outcome of AMF symbiosis for rice plants, partially by influencing the mycorrhizal P benefit. This finding has important implications for AMF application in rice production.

## Introduction

Arbuscular mycorrhizal fungi (AMF, subphylum Glomeromycotina; Spatafora et al., 2016) originated *ca*. 450 million years ago and today form a symbiosis with most vascular plant species, including many important crops. They have significant ecological functions in the movement of mineral nutrients (such as P and N) and water between soil and plants. Plants supply AMF with 10–30% of their photosynthetically fixed carbon (C) in exchange for a number of important nutrient feedbacks (especially P) from the fungal partner (Roth and Paszkowski, 2017). Nutritional exchange is a cornerstone of the interaction between AMF and plants and is essential for global C and P flow, as well as terrestrial ecosystem C sinks.

Arbuscular mycorrhizal fungi can also improve plants’ biotic resistance (soil-borne fungal and bacterial pathogens, nematodes, or root-chewing insects) (Rivero et al., 2018; Campo et al., 2020; but see Cosme et al., 2011; Bernaola et al., 2018b) and increase their tolerance under abiotic stress, including shading stress, drought, high salinity, and heavy metal contamination (Amir et al., 2019; Li et al., 2019), partly through triggering basal resistance in host plants (Cornejo et al., 2017). It is well known that pathogenic fungi can trigger an array of defense responses in plants in a process called basal resistance (Jones and Dangl, 2006; Boller and He, 2009). Pathogenesis-related (PR) proteins are widely used markers of plant defense responses (van Loon, 1999; van Loon et al., 2006). In rice, the induction of PR genes *PR1* and *PBZ1* is an indicator of the activation of the defense response to pathogen infection (Mitsuhara et al., 2008), as well as the colonization of AMF or other mutualists (Gutjahr et al., 2015; Tian et al., 2019). Rice plants can finely coordinate their responses to balance growth and defense through the modulation of plant hormones (Yang D. L. et al., 2012; Zipfel and Oldroyd, 2017). Considering the importance of developing disease-resistant crops and reducing fertilizer usage in modern agricultural production, knowledge of nutrient assimilation and disease resistance is of vital importance for plant breeding.

As one of the most important agricultural crops, rice (*Oryza sativa* L.) has been used as an important monocotyledon model plant for studies of arbuscular mycorrhizal (AM) symbiosis (Sawers et al., 2018; Wang et al., 2021). Globally, rice production is mostly conducted in wetland ecosystems characterized by anaerobic environments (FAO, 2011), where it was thought that AMF had difficulty forming symbiosis with rice plants. The results of studies of AMF in rice plants have been inconsistent. Several studies have reported that AMF are rare or absent in rice roots in flooded paddy fields (Ilag et al., 1987; Lumini et al., 2011). Other studies, meanwhile, have demonstrated the presence of AMF colonization inside rice roots in paddy fields (Vallino et al., 2009; Wang et al., 2015; Chen et al., 2017; Bernaola et al., 2018a), although flooding has generally exhibited a suppressive effect on AMF colonization in rice roots (Vallino et al., 2014; Zhang et al., 2015a). Our previous study found that AMF colonization in rice roots was commonly present at the heading and ripening stages, though absent or rare at the early growth stages (Wang et al., 2015), which could be partially explained by the well-developed aerenchyma, by which atmospheric oxygen can be delivered to rice roots at the heading and ripening stages (Wang et al., 2015). Some researchers have also reported the impact of AMF inoculation on rice plant growth under flooding (e.g., Secilia and Bagyaraj, 1994; Solaiman and Hirata, 1997; Bernaola et al., 2018a) and non-flooding conditions (e.g., Corrêa et al., 2014; Sánchez et al., 2015; Zhang et al., 2015b). The mycorrhizal growth response (MGR) of rice plants in these studies differed, ranging from positive to negative. Most studies reported that AMF increased rice plant biomass, grain yield, and P uptake under flooding conditions (Secilia and Bagyaraj, 1994; Solaiman and Hirata, 1996, 1997; Gewaily, 2019). By contrast, several others found that AMF inoculation resulted in a decrease in the amount of rice dry matter under flooded conditions (Solaiman and Hirata, 1995; Cosme et al., 2011; Bao et al., 2019). These studies provide important information about the ecological functions of AMF in paddy wetlands. However, their inconsistent results regarding the MGR of rice plants–which should be closely related to the complex environment of paddy fields and the different experimental conditions of the studies–pose an obstacle to a comprehensive understanding of the application prospects of AMF in rice-production systems (Xu et al., 2018; Bao et al., 2019). They also highlight the need to consider important influencing factors while assessing the functional role of AMF in paddy soils.

The interaction between rice plants and AMF is affected by a variety of biotic and abiotic factors, including rice cultivar, AMF identity, plant carbon level, irrigation regimes, fertilizer application, diseases, and insect pests (Solaiman and Hirata, 1997; Cosme et al., 2011). Among these factors, flooding may be an important driving force that regulates the response of plant performance to AMF symbiosis, because flooding can significantly affect root architecture and anatomy, as well as soil environmental factors, such as redox potential and pH (Vallino et al., 2014; Wang et al., 2016; Ding et al., 2019). Flooding can also affect the photosynthetic process and thus the C level of rice and other plants (Panda et al., 2008; Zhang et al., 2017), which is another important factor that may influence the mycorrhizal performance of rice in paddy fields (Korhonen et al., 2004; Konvalinková et al., 2015). Indeed, previous studies have shown that low plant C levels (e.g., shading) suppress AMF symbiosis due to the increased C competition between plants and AMF (Konvalinková et al., 2015; Konvalinková and Jansa, 2016). Shading can also affect the MGR by influencing P uptake in host plants (Sulaiman and Sadiq, 2020), and even short periods of shade could have important consequences for the functioning of mycorrhizal symbiosis in terms of P transfer between the fungus and the plants (Konvalinková et al., 2015). Therefore, to survey the potential effects of flooding on AMF symbiosis, it is important to consider the C level of host plants. In the context of global climate change, increased rainfall and decreased hours of sunshine during the rice growth period are widespread in rice-production areas (Chou et al., 2019; Ray et al., 2019), and heavy haze and aerosol pollution may further decrease solar irradiance (Tie et al., 2016). Surprisingly, although the rice mycorrhizal response under varied flooding regimes has been well documented (Sánchez et al., 2015; Bao et al., 2019), the impact of plant C levels on rice mycorrhizal responses to flooding has, to our knowledge, not been reported yet. Against the background of a rising global population and increased environmental pollution, surveying the AMF and rice interaction in wetland habitats is important for understanding their ecological functions as well as the AMF application potential in agricultural production systems.

Here, we aimed to survey the regulatory effects of flooding on the mycorrhizal responses of rice plants with or without shading stress. Specifically, we investigated the following two questions: (i) How does the MGR of rice plants respond to flooding regimes? and (ii) Does the effect of flooding on MGR vary at different plant C levels [as assessed by the application (or not) of shading to suppress plant photosynthetic activity (Yang et al., 2019)]? The rice-growth response to AMF symbiosis was recorded in Exp. 1 using two rice varieties at two rice growth stages under three different flooding intensities. The potential mechanisms involved in the different growth responses to different flooding and shading treatments were assessed in Exp. 2. This study can provide guidance for optimizing rice grain yield in sustainable agriculture and support the further application of AMF fertilizer in practical agricultural production.

## Materials and Methods

### Experiment Design

Two greenhouse experiments with randomized complete block designs are reported in this paper. Exp. 1 surveyed the AMF responses of rice growth under different flooding intensities. It consisted of four treatment factors, with a full factorial design: AMF inoculation (with and without AMF [the AM and non-inoculated (NM) treatments, respectively)], rice cultivar [Guinongzhan (GNZ) and Sanhuangzhan (SHZ)], harvest stage (heading and mature), and flooding intensity [non-flooding, intermittent flooding (IF), and continuous flooding (CF)]. The rice cultivar GNZ (but not SHZ) has previously been characterized with a relatively high O_2_ supply to the rhizosphere (i.e., radial oxygen loss) *via* well-developed aerenchyma (Wu et al., 2011). Each treatment was replicated three times, with a total of 72 pots. Plants were grown in a climate-controlled greenhouse, with a 16/8-h light/dark cycle. The average photosynthetic photon flux density during the daytime was 690 μmol m^–2^s^–1^ based on three measurements with an Illuminometer (UNI-T UT383, China). Half of the pots were inoculated with a 6% *Rhizophagus irregularis* inoculum (v/v, AM), and the rest were left NM. Before being subjected to different flooding treatments, rice plants were cultivated for 30 days under non-flooding conditions in which the soil surface was wet but not water-logged, to ensure successful AMF colonization in rice roots based on results from our pilot experiment (under a flooding environment, the AMF colonization intensity in rice seedlings was usually lower than 5%, unpublished data) and other studies (e.g., Solaiman and Hirata, 1997). Rice plants intended for non-flooding and continuous-flooding treatments were grown in drained (but wet) and flooded/waterlogged soils (*ca*. 1–2 cm of water above soil), respectively. Plants were grown alternately under drained and flooded conditions every 10 days to simulate IF (Bao et al., 2019).

In Exp. 2, we further explored the involved mechanisms underlining the observed differences in the effects of AMF on rice plants among different flooding regimes in Exp. 1, using the GNZ cultivar. There were three treatment factors in Exp. 2: AMF inoculation (AM and NM), plant C level [through application of shading (or not) to suppress plant photosynthesis activity (Yang et al., 2019)], and flooding intensity (non-flooding and IF). Each treatment was replicated five times, with a total of 40 pots. Plants were grown in a climate-controlled greenhouse, with a 16/8-h light/dark cycle and an average photosynthetic photon flux density of 690 μmol m^–2^s^–1^. Shading treatment was accomplished by use of a black nylon net that prevented *ca.* 60% light (average light intensity was *ca.* 280 μmol m^–2^s^–1^), under which rice should have been carbon stressed (Venkateswarlu et al., 1987). The application of flooding (non-flooding and IF) and AMF (NM and AM) treatments were as described in Exp. 1.

### Biological Materials and Growth Conditions

Inoculum of *Rhizophagus irregularis* (formerly *Glomus intraradices*), a model AMF species with available genome information (Tisserant et al., 2014) that is frequently found in paddy wetlands (Vallino et al., 2009; Lumini et al., 2011; Wang et al., 2015), was provided by MycAgro Lab, France.^1^ It was produced by propagation on *Plantago lanceolata* L. in pots filled with sterilized quartz sand, and the fungal inoculum was a mixture of spores (>15 per milliliter inoculum), mycelium, sandy soil, and root fragments.

Seeds of *Oryza sativa* L. cv. Guinongzhan and Sanhuangzhan (SHZ) are commonly planted indica rice cultivars in southern China. They were surface-sterilized in a 70% (v/v) solution of alcohol for 15 min and pre-germinated on moist filter paper for about 48 h at 30°C until the appearance of radicles. The germinated seeds were then planted in the pots (12 cm in height, 1 L in volume, three seedlings per pot) containing 800 ml substrates with or without AMF inoculation (6% inoculum by volume). The substrate consisted of a mixture of 75% soil and 25% washed quartz sand. The soil was collected from a paddy field in southern China and had the following characteristics: pH 7.76, total P 0.38 g kg^–1^, total N 1.61 g kg^–1^, available P 33.8 mg kg^–1^, and available N 90.4 mg kg^–1^. The field soil and washed quartz sand were sterilized three times (121°C for 20 min each time) in order to eliminate indigenous AMF. The NM control received autoclaved inoculum plus a microbial wash obtained by passing the suspensions of inoculum through a paper filter (particle retention: 11 μm).

The pots were maintained in the greenhouse with a temperature of 30/26°C (day/night) and 60% relative humidity until harvest. Plants were watered every day with deionized water and supplied with 20 ml Kimura B solution (Ma and Takahashi, 1990) with half (Exp. 1) and one-fourth (Exp. 2) of the normal P concentration (45 μM KH_2_PO_4_) every 2nd week. In Exp. 1, rice plants were harvested at the heading stage (day 65) and mature stage (day 95); in Exp. 2, rice plants (GNZ cultivar) were harvested at the heading stage. During harvest, rice plants were carefully washed with tap water and then deionized water. Shoots were cut at the hypocotyl-root boundary and were subsequently dried at 70°C. A small aliquot of roots, including crown roots, large lateral roots, and fine lateral roots, was randomly selected and stored at −80°C for real-time quantitative PCR. A small portion of the roots was used for AMF colonization intensity qualification, while the rest of the roots were dried, weighed, and used for P measurement.

### Qualification of Arbuscular Mycorrhizal Fungi Colonization Intensity and Extraradical Hyphal Density

Root samples collected at both the heading and maturing stages (Exp. 1 and Exp. 2) were used for the assessment of mycorrhizal colonization. They were treated with 10% w/v KOH at 90°C for 10–20 min and then stained using trypan blue. The AMF colonization rates of vesicles, arbuscules, and hyphae (i.e., root-colonizing structures of AMF other than vesicles and arbuscules) were quantified using the line-intersect method (McGonigle et al., 1990). For each individual sample, at least 100 intersects from 20 root segments (1 cm in length) were scored under a light microscope (Carl Zeiss, Axiostar Plus, Jena, Germany) at 400 × magnification.

The density of extraradical hyphae, by which AMF absorb and deliver mineral nutrients to host plants (Smith and Read, 2010), in the rhizosphere soil of different treatments from Exp. 2 (but not Exp. 1, in which the shading treatment was not applied) was analyzed to roughly estimate the AMF abundance and functional capability in soil under different flooding and shading treatments. Extraradical hyphae were extracted from a 0.1 g air-dried soil sample using 18 ml 20 g/L sodium hexametaphosphate, then filtered through 20 μm mesh, stained with trypan blue overnight, and collected by filtering through 1.2 μm nitrocellulose filters. Extraradical hyphal density (EHD) was quantified *via* the gridline intersect method at 100 × magnification [modified from Miller et al. (1995)].

### Measurement of Chlorophyll Fluorescence Parameters, Biomass, and Plant Phosphorus Level

Changes in chlorophyll fluorescence emission arising mainly from the PS II provide information on almost all aspects of photosynthetic activity (Zhang et al., 2018). The modulated chlorophyll fluorescence measurements were taken by PAM-2500 (WALZ Company, Germany) from leaves from growing rice plants 1 or 2 days before harvest at the heading and maturing stages. Fluorescence was measured in the dark-adapted state (the leaves were kept in darkness for at least 20 min). The detailed measurement methods for effective PS II quantum yield [Y(II)], maximal PS II quantum yield [Fv/Fm = Y(II)max], electron transport rate (ETR), and the yield of non-photochemical quenching [Y(NPQ)] referred to the manufacturer’s instructions (Schreiber et al., 2011). These parameters can be used as a measure of the overall photosynthetic activity, i.e., plant C assimilation (Kalaji et al., 2014, 2017).

For plant phosphorus measurement, dried samples (leaf, stem, and root) were milled to fine powder and sieved through a 1.0 mm mesh. They were then digested with concentrated HNO_3_ and H_2_O_2_ (5:1, v/v) by a microwave digestion system (WX-8000, Shanghai, China). Total P levels were determined by a molybdenum blue colorimetry method according to Watanabe and Olsen (1965).

### RNA Extraction, cDNA Synthesis, and Real-Time Quantitative PCR

Total RNA was extracted from rice roots (∼1 g in fresh weight, GNZ, harvested at heading stage) using the Trizol method (Takara, Japan), according to the manufacturer’s instructions. RNA quality was assayed on an Agilent 2100 Bioanalyzer (Agilent Technologies, United States). Genomic DNA elimination reaction and single-strand cDNA synthesis were performed using a PrimeScript^TM^ RT reagent Kit (Takara, Japan). Quantitative real-time PCR (qRT-PCR) was carried out using an SYBR^®^ Premix Ex Taq^TM^ Kit (Takara, Japan) in a QuantStudio^TM^ 6 Flex Real Time PCR system (Applied Biosystems, United States), according to the following program: 50°C for 2 min, 95°C for 30 s, followed by 40 cycles of 95°C for 5 s, 55°C for 20 s, 72°C for 20 s, and a melting curve (60–95°C at a heating rate of 0.1°C per s) was recorded at the end of each run to exclude the non-specific PCR amplification. Three biological and three technical replicates were used for each sample, and the comparative 2^–ΔΔCt^ method was used to quantify the relative specific mRNA levels of each reaction (Livak and Schmittgen, 2001). For quality control, only Ct values from three technical replicates leading to a Ct mean with a standard deviation below 0.5 were considered (Pérez-Tienda et al., 2011). Expression profiles of P transporter marker genes (*OsPT2* and *OsPT6* for the direct pathway, and *OsPT11* and *GintPT* for the mycorrhizal pathway, see Yang S.-Y. et al., 2012; Bao et al., 2019) were analyzed in order to characterize the mycorrhizal effect on rice P acquisition. In addition, two PR genes, *OsPR1* and *OsPBZ1*, were also analyzed to reveal the basal defense status of rice plants under different treatments (Gutjahr et al., 2015; Tian et al., 2019). Transcript levels were normalized to the Ct value of *OsCyclophilin2* for the plant P transporter marker genes *OsPT2*, *OsPT6*, and *OsPT11* (Yang D. L. et al., 2012), plant defense marker genes *OsPR1*, *OsPBZ1* (Mitsuhara et al., 2008), and *GintEF1*α (González-Guerrero et al., 2010) for the mycorrhizal pathway AMF P transport genes *GintPT* (Fiorilli et al., 2013) (see Supplementary Table 1 for primer information).

### Statistical Analysis

The AMF percentage colonization data were (arcsine x)^1/2^ transformed to achieve homogeneity of variances before an analysis of variance (ANOVA) was performed. MGR, mycorrhizal yield response (MYR), and mycorrhizal P response (MPR) were calculated (Corrêa et al., 2014) based on the plant biomass, yield (grain weight), and plant P content, respectively, following the formula below:


MR%=(AM-NM)/NM×100%,

where, AM is the observed value for the target parameter (biomass for MGR, yield for MYR, and P content for MPR) in mycorrhizal plants, and NM represents corresponding values from non-mycorrhizal plants.

Data normality was determined using the Kolmogorov–Smirnov test, and ANOVA was conducted following different parametric tests depending on the tests for homogeneity of variance. A three-way ANOVA was applied to test the effects of flooding intensity, growth stage, and rice cultivar on AMF parameters in Exp. 1, and to analyze the effects of AMF inoculation, flooding, and shading treatments on rice parameters in Exp. 2. A two-way ANOVA was used to test the effects of flooding and shading treatments on AMF parameters. A one-way ANOVA followed by a Duncan test was used to analyze differences in AMF parameters and rice parameters. Variation partitioning analyses (VPA) were conducted to examine the main driving factors of the rice biomass using CANOCO 5.0 (Microcomputer Power, Ithaca, NY, United States). We classified the environmental factors into three groups in Exp. 1 (AM status, rice cultivar, and flooding) and in Exp. 2 (AM status, flooding, and shading). Additionally, general linear models were performed to test the correlation between MGR and MPR. All statistical analyses were performed using SPSS 20.0 software (SPSS Inc., Chicago, IL, United States).

## Results

### Arbuscular Mycorrhizal Fungi Colonization Intensity

Typical AMF structures, including arbuscules (colonization rate: 26.2% ± 13.5%), vesicles (12.7% ± 12.0%), and hyphae (20.1% ± 15.9%), were detected in all mycorrhizal plants (Supplementary Figure 1), and no mycorrhizal colonization was found in non-mycorrhizal controls (Tables 1, 2). In both Exps. 1 and 2, flooding exerted significant effects on the AMF colonization rates (*P* < 0.01, Table 3 and Supplementary Table 2), and the root colonization rates of all typical AMF structures and EHD were mostly significantly lower with increased flooding intensity (*P* < 0.05). In Exp. 1, the root colonization rates of GNZ and SHZ were decreased by 41.0–81.9% and 26.2–91.8%, respectively, following flooding regimes. In Exp. 2, the root colonization rates were decreased by 28.0–66.4% and 39.6–68.2% upon flooding treatment under non-shading and shading conditions, respectively (Tables 1, 2).

**TABLE 1:** Arbuscular mycorrhizal fungi (AMF) colonization intensity in the rice roots collected at the heading and mature stages from none, intermittent, and continuous flooding (NF, IF, and CF, respectively) conditions.

**Variety**	**Flooding**	**Stage**	**TC%**	**AC%**	**VC%**	**HC%**
GNZ	NF	Heading	28.1 ± 2.4 de	21.7 ± 2.9 ef	17.7 ± 0.2 b	17.7 ± 0.2 cd
	IF		14.7 ± 2.9 fg	12.8 ± 1.4 f	4.7 ± 4.7 c	4.7 ± 4.7 de
	CF		20.7 ± 0.4 ef	20.0 ± 0.5 ef	3.2 ± 3.2 c	3.2 ± 3.2 e
	NF	Maturing	61.0 ± 1.2 a	50.1 ± 3.2 a	36.2 ± 1.9 a	38.9 ± 1.9 b
	IF		41.8 ± 2.9 bc	36.5 ± 1.7 bc	10.3 ± 6.4 bc	20.0 ± 3.3 c
	CF		32.1 ± 1.3 cd	27.4 ± 2.7 cde	11.2 ± 1.5 bc	13.9 ± 4.1 cde
SHZ	NF	Heading	57.9 ± 6.8 a	40.4 ± 4.4 b	18.0 ± 2.0 b	54.1 ± 8.2 a
	IF		6.7 ± 3.3 g	3.3 ± 3.3 g	3.3 ± 3.3 c	6.7 ± 3.3 cde
	CF		15.5 ± 3.6 fg	13.3 ± 3.3 f	3.3 ± 3.3 c	12.2 ± 6.5 cde
	NF	Maturing	44.6 ± 4.1 b	32.8 ± 0.5 bcd	29.9 ± 4.0 a	34.0 ± 3.2 b
	IF		29.1 ± 2.9 de	24.2 ± 2.3 de	8.6 ± 4.8 bc	16.3 ± 3.6 cde
	CF		35.4 ± 4.2 bcd	31.8 ± 5.8 bcd	6.4 ± 6.4 bc	19.0 ± 1.7 c

*Rice varieties are indicated as GNZ, Guinongzhan; SHZ, Sanhuangzhan. TC%, total colonization [calculated as the occurrence frequency of AMF structures (vesicles, arbuscules, or hyphae)]; AC%, arbuscular colonization; VC%, vesicle colonization; and HC%, hyphal colonization. Values with different letters in the same column are significantly different at the 0.05 probability level (mean ± SE, *n* = 3).*

**TABLE 2:** Arbuscular mycorrhizal fungi colonization intensity and extraradical hyphal density (EHD, m g^–1^) in rice roots of Guinongzhan collected at the heading stage from different flooding (NF, non-flooding, IF, intermittent flooding) and shading (S, shading, NS, non-shading) conditions.

**Flooding**	**Shading**	**TC%**	**AC%**	**VC%**	**HC%**	**EHD**
NF	NS	64.9 ± 4.0 a	37.5 ± 6.2 a	55.7 ± 4.8 a	65.3 ± 5.0 a	13.9 ± 3.1 a
	S	53.0 ± 2.9 b	23.9 ± 6.3 ab	53.0 ± 2.9 a	50.5 ± 4.1 b	3.2 ± 0.6 b
IF	NS	42.0 ± 3.2 c	12.6 ± 2.0 b	40.1 ± 4.2 b	31.4 ± 2.6 c	0.3 ± 0.09 b
	S	32.0 ± 2.2 c	7.6 ± 4.1 b	30.7 ± 1.8 b	27.9 ± 2.6 c	0.5 ± 0.2 b

*TC%, total colonization [calculated as the occurrence frequency of AMF structures (vesicles, arbuscules, or hyphae)]; AC%, arbuscular colonization; VC%, vesicle colonization; HC%, hyphal colonization. Values with different letters in the same column are significantly different at the 0.05 probability level (mean ± SE, *n* = 3).*

**TABLE 3:** Analysis of parameter estimates from generalized linear models: effects of AMF status, flooding, and shading treatments on tested plants and AMF parameters.

**Variables**	**AMF status**		**Flooding**		**Shading**	
	**F**	**Sig.**	**F**	**Sig.**	**F**	**Sig.**
**Growth parameters**						
Plant biomass	6.63	*	13.1	***	150	***
MGR	–	–	0.00	ns	10.1	**
**Plant C assimilation**						
Fv/Fm	6.22	*	2.30	ns	5.79	*
Y(II)	126	***	218	***	34.1	***
ETR	127	***	214	***	33.3	***
Y(NPQ)	112	***	280	***	6.17	**
**AMF structures**						
VC%	–	–	27.2	***	2.79	ns
HC%	–	–	57.5	***	6.05	*
AC%	–	–	12.9	**	2.03	ns
TC%	–	–	48.4	***	12.1	**
EHD	–	–	25.0	***	10.6	**
**AMF P delivery to plants**						
*OsPT11*	–	–	3.10	ns	14.8	**
*GintPT*	–	–	0.34	ns	2.26	ns
*OsPT2*	61.1	***	3.22	ns	1.40	ns
*OsPT6*	20.3	***	5.01	*	0.44	ns
MPR	–	–	0.49	ns	38.6	***
Shoot P conc.	14.9	***	292	***	7.88	**
Root P conc.	0.08	ns	5.85	*	5.85	*
**Plant defense system**						
*OsPR1*	1.10	ns	0.00	ns	34.1	***
*OsPBZ1*	31.2	***	13.0	**	14.4	**

The chlorophyll fluorescence parameters [Fv/Fm, Y(II), ETR, and Y(NPQ)] are indicative of plant C assimilation; *OsPT2* and *OsPT6* expression levels are indicative of the direct P uptake pathway; *OsPT11* and *GintPT* expression levels are indicative of the mycorrhizal P uptake pathway; *OsPR1* and *OsPBZ1* expression levels are indicative of rice basal defense conditions. MGR, mycorrhizal growth response; MPR, mycorrhizal P response; Fv/Fm, maximum quantum yield; Y(II), actual quantum yield; ETR, electron transport rate, Y(NPQ), the yield of non-photochemical quenching. VC, HC, AC, and TC% indicate vesical, hyphal, arbuscular, and total colonization intensities, respectively; EHD, extraradical hyphal density; ns, not significant at the 0.05 probability level; *, **, and *** represent statistical significance at the 0.05, 0.01, and 0.001 probability levels, respectively.

Shading also had significant effects on both AMF colonization intensity (HC and TC%, *P* < 0.05) and EHD (*P* < 0.01, Table 3). It decreased the AMF colonization intensity of rice plants by 4.9–36.3% and 11.2–39.7% under non-flooding and flooding conditions, respectively, though the differences between shading and non-shading treatments were usually not statistically significant (Table 2). The EHD in both flooded and non-flooded soils were both decreased by *ca.* 70% upon shading treatment (Table 2), and under non-flooding conditions, the differences in EHD were statistically significant between shading and non-shading treatments (*P* < 0.05).

The rice growth stage showed significant effects on AMF colonization rates (*P* < 0.01; Supplementary Table 2). In the GNZ cultivar, there were usually significantly higher AMF colonization rates in the roots from the mature stage than in those from the heading stage (*P* < 0.05, Table 1), while in the SHZ cultivar the AMF colonization intensities between the heading and maturing stages were generally at a similar level (Table 1). At the heading stage the GNZ cultivar had significantly lower AMF arbuscular, hyphal, and total colonization rates than SHZ (*P* < 0.05), while at the maturing stage the GNZ cultivar had higher arbuscular and total colonization rates than SHZ in the non-flooding and intermittent-flooding treatments (*P* < 0.05, Table 1).

### Mycorrhizal Responsive Patterns

**FIGURE 1:**
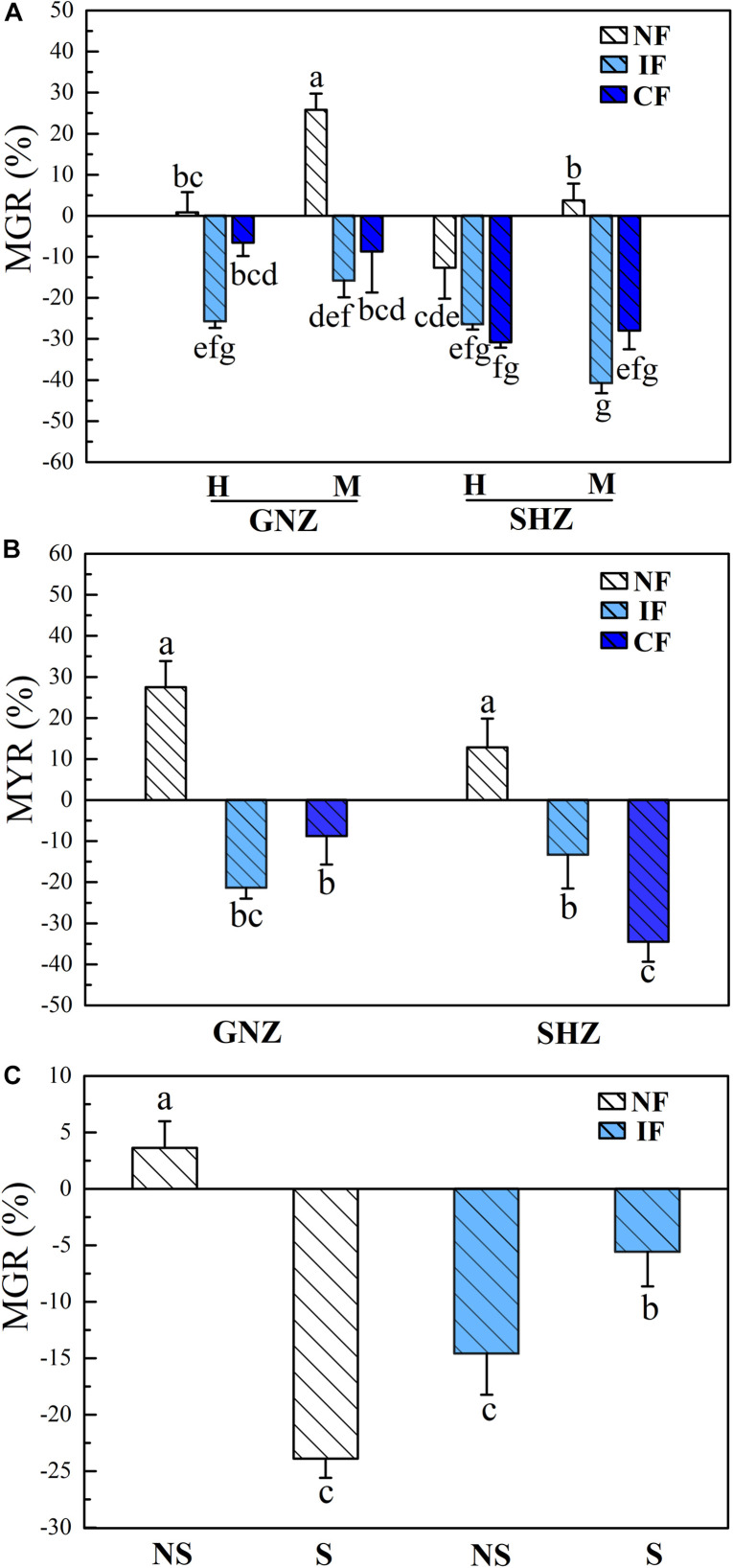
Mycorrhizal growth response [MGR, **(A)**] and mycorrhizal yield response [MYR, **(B)**] in Exp. 1, as well as MGR **(C)** in Exp. 2. Rice varieties are indicated as GNZ, Guinongzhan; and SHZ, Sanhuangzhan. The rice varieties GNZ and SHZ were used in Exp. 1, and GNZ was used in Exp. 2. Rice growth stages are indicated as H, Heading; and M, Maturing. Rice plants were harvested at the heading and maturing stages in Exp. 1 and harvested at the heading stage in Exp. 2. Flooding treatments are indicated as NF, Non-flooding; IF, Intermittent flooding; and CF, Continuous flooding. Shading treatments are indicated as NS, Non-shading; and S, Shading. Different letters above the columns indicate significant differences at the *P* < 0.05 level. Means and standard errors from three **(A)** and five replicates **(B)** are shown.

In Exp. 1, AMF colonization (explained 8.0% of variance) and flooding intensity (explained 14.8% of variance) both had significant effects on the biomass and grain weight of rice plants (*P* < 0.05, Supplementary Table 2), and the effects of AMF colonization on rice performance varied under different flooding conditions. Under non-flooding conditions, AMF colonization showed no significant effects on rice biomass and grain yield, except that for the GNZ cultivar AMF colonization significantly increased the biomass of rice from the maturing stage (*P* < 0.05, Supplementary Figures 2A,B). For both GNZ and SHZ, the MGR at the heading stage (GNZ: 1.9%, SHZ: −11.8%) was correspondingly significantly lower than that at the maturing stage (GNZ: 24.3%, SHZ: 3.6%, *P* < 0.05), and their MYRs were 27.5 and 12.8%, respectively (Figures 1A,B). Under IF and CF conditions, AMF showed evident negative effects on the growth and grain weight for both the GNZ (6.6–25.8% reduction of plant biomass, and 8.8–21.4% reduction of grain weight) and SHZ cultivars (26.5–40.8% reduction of plant biomass, and 13.4–34.6% reduction of grain weight), and the MGR and MYR values of flooded rice (IF and CF) were both significantly lower than those in the corresponding non-flooding treatment (*P* < 0.05, Figures 1A,B). The GNZ and SHZ cultivars showed a very similar responsive pattern to AMF inoculation under all treatments, although AMF had a greater inhibitive effect on the growth of SHZ compared to that of GNZ (*P* < 0.05, Figures 1A,B). For both GNZ and SHZ, flooding generally significantly increased the biomass and grain weight of rice plants (Supplementary Figures 2A,B).

Consistent with the results from Exp. 1, in Exp. 2 AMF colonization and flooding both showed significant effects on rice (GNZ) biomass (*P* < 0.05, Table 3); also, the positive mycorrhizal response of rice biomass under non-flooding conditions (3.6%) became negative (−14.6%) under flooding conditions (Figure 1C). Flooding significantly increased the biomass of rice plants under shading environments (*P* < 0.05, Supplementary Figure 2C). The MGR in non-flooded rice was significantly higher than that cultivated in the flooded environment (*P* < 0.05, Figure 1C).

Shading significantly decreased plant biomass (*P* < 0.001, Table 3), and the MGRs of rice biomass were negative under both non-flooding (−23.9%) and flooding (−5.6%) conditions. The variance portioning analysis showed that shading exerted much stronger impacts on rice biomass (explained 71.6% of variance) than flooding (6.4%) and AMF status (2.3%). Shading significantly decreased the MGR of rice under non-flooding conditions (from 3.6 to −23.9%) while it significantly increased MGR under IF conditions (from −14.6 to −5.6%) (*P* < 0.05, Figure 1C).

### Chlorophyll Fluorescence Parameters

**FIGURE 2:**
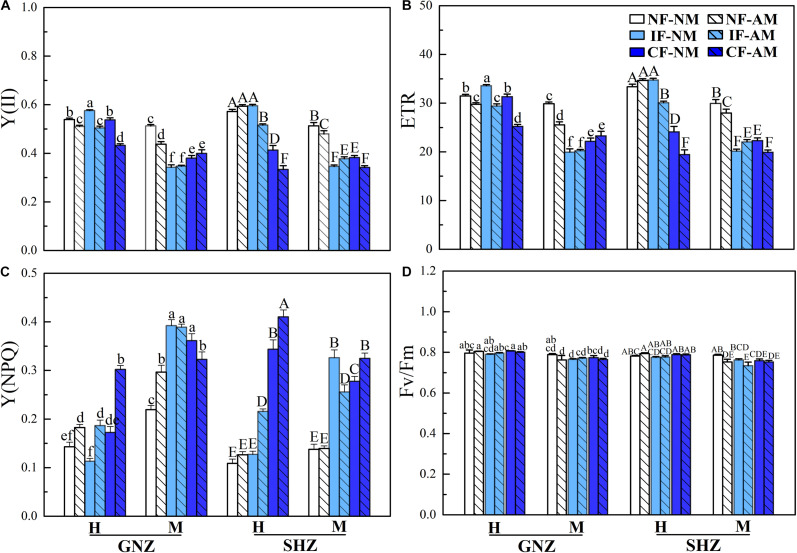
Rice chlorophyll II fluorescence parameters under non-flooding (NF), intermittent flooding (IF), and continuous flooding (CF) conditions in Exp. 1. **(A)** Actual quantum yield [Y(II)], **(B)** the electron transport rate (ETR), **(C)** the yield of non-photochemical quenching [Y(NPQ)], and **(D)** the maximum quantum yield (Fv/Fm) in the mycorrhizal (AM) and non-mycorrhizal (NM) leaves of Guinongzhan (GNZ) and Sanhuangzhan (SHZ) collected at heading (H) and maturing (M) harvest stages. Lowercase letters are used in GNZ cultivar analysis, and uppercase letters are used in SHZ cultivar analysis. Different letters above the columns indicate significant differences at the *P* < 0.05 level. Means and standard errors from three replicates are shown.

In both Exp. 1 and Exp. 2, the chlorophyll fluorescence parameters were significantly affected by AMF inoculation, rice growth stage, and flooding intensity (*P* < 0.05, Table 3 and Supplementary Table 2). In both experiments, AMF colonization significantly decreased the effective PS II quantum yield [Y(II)] (Figure 2A and Supplementary Figure 2A) and ETR (Figure 2B and Supplementary Figure 2B) in most cases. By contrast, AMF colonization increased the Y(NPQ) in most cases (Figure 2C and Supplementary Figure 2C). These outcomes were observed regardless of flooding and shading environments (Figure 2 and Supplementary Figure 2). In Exp. 1, there were higher Y (II) (Figure 2A) and ETR (Figure 2B) and lower Y(NPQ) (Figure 2C) in non-flooding conditions in comparison to both flooding treatments (*P* < 0.05) except for Y (II) and ETR at the heading stage and the Y(NPQ) of the GNZ cultivar at the mature stage. Rice plants in the heading stage generally had higher Y (II) and ETR and lower Y(NPQ) than plants at the mature stage. The maximal PS II quantum yield (Fv/Fm) value was significantly decreased in rice plants from the heading to mature stages (*P* < 0.05, Figure 2D), and was not affected by AMF inoculation, rice variety, flooding, and shading treatments (*P* > 0.05, Figure 2D and Supplementary Figure 2D). In Exp. 2, AMF inoculation, shading, and flooding all significantly decreased Y (II) (Supplementary Figure 3A) and ETR (Supplementary Figure 3B) and increased Y(NPQ) (*P* < 0.05) (Supplementary Figure 3C).

### Effects of Arbuscular Mycorrhizal Fungi Symbiosis on Rice P Acquisition

**FIGURE 3:**
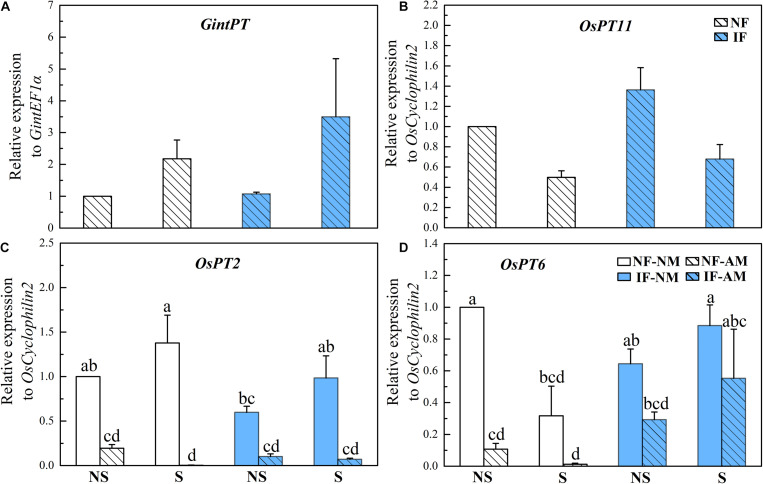
Relative expression profiles of the P delivery marker genes from arbuscular mycorrhizal fungi (AMF) [**(A)**: *GintPT*] and rice [**(B)**: *OsPT11*, **(C)**: *OsPT2*, **(D)**: *OsPT6*,] and in the mycorrhizal (AM) and NM roots under different flooding (IF, intermittent flooding, NS, non-flooding) and shading (S, shading, NS, non-shading) conditions in Exp. 2. Guinongzhan (GNZ) was used, and rice plants were harvested at the heading stage. The expressions of rice and AMF genes are relative to the expression of the rice house-keeping gene *OsCyclophilin2*. Different letters above the columns indicate significant differences at the *P* < 0.05 level. Means and standard errors from three replicates are shown.

*GintPT* and *OsPT11*, which are indicative of the mycorrhizal P pathway in rice roots, were both detected in AM plants but not in NM controls (Figures 3A,B), suggesting that AMF colonization activated the mycorrhizal P pathway under all flooding and/or shading conditions. In rice plants inoculated with AMF, shading did not influence the relative expression level of *GintPT* (*P* > 0.05, Table 3), while it significantly decreased the relative expression level of *OsPT11* (*P* < 0.01). By contrast, flooding had no significant effect on the relative expression levels of *OsPT11* and *GintPT* (*P* > 0.05, Table 3). The expression levels of the direct P pathway marker genes *OsPT2* and *OsPT6* were significantly influenced by AMF inoculation (*P* < 0.001) but not by flooding and shading treatments (*P* > 0.05, Table 3). There were lower expression levels of *OsPT2* (by 80.5–99.8%) and *OsPT6* (by 37.5–96.1%) in AM roots than in NM roots (*P* < 0.05, Figures 3C,D), indicating that AMF colonization suppressed the direct P pathway in rice roots. Neither shading nor flooding treatments had significant effects on the *OsPT2* and *OsPT6* expression levels (*P* > 0.05, Figures 3A,B).

**FIGURE 4:**
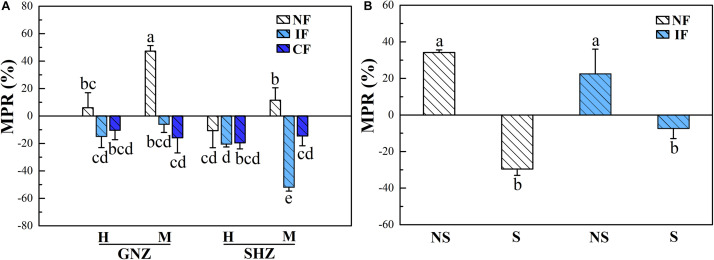
Mycorrhizal phosphorus response (MPR) in Exp. 1 **(A)** and Exp. 2 **(B)**. Rice varieties are indicated as GNZ, Guinongzhan; SHZ, Sanhuangzhan. The rice varieties of GNZ and SHZ were used In Exp. 1, and SHZ was used in Exp. 2. Rice growth stages are indicated as H, Heading; and M, Maturing. Rice plants were harvested at the heading and maturing stages in Exp. 1 and harvested at the heading stage in Exp. 2. Flooding treatments are indicated as NF, Non-flooding; IF, Intermittent flooding; CF, Continuous flooding. Shading treatments are indicated as NS, Non-shading; S, Shading. Different letters above the columns indicate significant differences at the *P* < 0.05 level. Means and standard errors from three **(A)** and five replicates **(B)** are shown.

In Exp. 1, AMF colonization (*P* < 0.01) had significant effects on the shoot P level (concentration and content), and the effects of AMF varied under different flooding regimes, as indicated by the significant effects of flooding intensity on shoot MPR (*P* < 0.001, Figure 4A and Supplementary Table 2). Under non-flooding conditions, AMF colonization significantly increased the shoot P content of GNZ rice harvested at the maturing stage (MPR = 47.2%, *P* < 0.05), while it showed no significant effects on the shoot P level of other rice plants (*P* > 0.05, Supplementary Figures 4A,B). Under intermittent- and continuous-flooding conditions, the MPRs in GNZ and SHZ were both significantly lower than those in the corresponding non-flooding treatments at the maturing (but not heading) stage (*P* < 0.05, Figure 4A). AMF colonization decreased the shoot P contents for both flooded rice of GNZ (by 6.1–15.9%) and SHZ (by 14.7–52.0%), though the inhibitory effect was significant only in the maturing SHZ rice under IF conditions (*P* < 0.01, Supplementary Figure 4B). The harvesting stage also exerted significant effects on shoot P level (*P* < 0.001). At the heading stage, the shoot P concentration and content of rice plants were significantly higher than those at the maturing stage (*P* < 0.05, Supplementary Figures 4A,B). The correlation analysis showed that MPR and MGR were highly positively correlated (*r* = 0.889, *P* = 0.000, Supplementary Figure 5A). The MPR and MGR were both positively correlated with the total colonization rates (*P* < 0.01, Supplementary Figures 6A,B).

In Exp. 2, AMF colonization significantly affected the shoot P concentration of rice (*P* < 0.001, Figure 3), and its effect was significantly influenced by the shading (*P* < 0.01) and flooding (*P* < 0.001) treatments (Table 3). Under the non-shading treatment, AMF significantly increased the shoot P concentration of rice plants in both non-flooding (by 21.1%) and IF (by 32.6%) environments (*P* < 0.05), while under shading conditions AMF colonization significantly decreased the shoot P concentration (by 11.8%) in the non-flooding environment (*P* < 0.05, Supplementary Figure 4C). Under both non- and intermittent-flooding regimes the MPR of shaded rice (non-flooding: −29.6%, intermittent-flooding: −7.4%) was correspondingly significantly lower than that of the non-shaded plants (non-flooding: 34.2%, intermittent-flooding: 22.5%, *P* < 0.05, Figure 4B). The correlation analysis showed that MPR was strongly positively correlated with MGR (*r* = 0.849, *P* = 0.000, Supplementary Figure 5B) and the EHD in soil (*r* = 0.525, *P* = 0.008, Supplementary Figure 6C).

### Effect of Arbuscular Mycorrhizal Symbiosis on Expression of Defense-Related Genes

**FIGURE 5:**
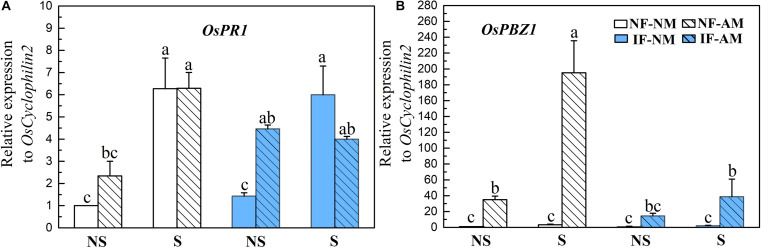
Relative expression levels of defense marker genes *OsPR1* **(A)** and *OsPBZ1* **(B)** in the mycorrhizal (AM) and NM rice roots under different flooding (IF, intermittent flooding, NS, non-flooding) and shading (S, shading, NS, non-shading) conditions in Exp. 2. Guinongzhan (GNZ) was used, and rice plants were harvested at the heading stage. Expression of rice genes are relative to the rice house-keeping gene *OsCyclophilin2*. Different letters above the columns indicate significant differences at the *P* < 0.05 level. Means and standard errors from three replicates are shown.

The expression levels of the common defense marker genes *OsPR1* and *OsPBZ1* were significantly influenced by shading (*P* < 0.01, Table 3), and *OsPBZ1* was significantly affected by AMF status (*P* < 0.01) and flooding intensity (*P* < 0.01). The *OsPR1* (0.7–3.1 fold) and *OsPBZ1* (16–60 fold) were significantly higher in AM plants than that in NM controls, except for the *OsPR1* expression under shading conditions and the *OsPBZ1* expression under non-shading plus IF conditions (*P* < 0.05, Figures 5A,B), indicating the activation of the common defense system by AMF colonization. In NM roots, the common defenses were not changed by flooding treatments, while in AM roots, the *OsPBZ1* expression level was decreased fivefold by flooding in shading conditions (*P* < 0.05, Figure 5). In NM roots, the expression level of *OsPR1*, but not *OsPBZ1*, was increased fourfold to sixfold by shading treatment (*P* < 0.05, Figure 5). In AM roots, shading also significantly increased the relative expression levels of *OsPR1* (+270%) and *OsPBZ1* (+560%) under NF conditions (*P* < 0.05, Figure 5).

## Discussion

### Mycorrhizal Growth Response of Rice Under Different Flooding and Shading Regimes

Arbuscular mycorrhizal fungi are important components in paddy fields and some other wetlands (Wang et al., 2010, 2015), but their functional role in paddy wetlands is not fully explained yet (Bao et al., 2019). Vallino et al. (2014) showed that rice flooding negatively affects root branching and AMF colonization but not fungal viability. In our study, the results of molecular and physiological analyses confirmed the establishment of active and functional AM symbiosis in rice plants under all flooding and/or shading environments. The expression file of *GintPT* suggested that AMF activity per unit was not suppressed by flooding and/or shading treatments. This study’s findings further support the concept that AMF are important functional components in wetlands, even in the absence of sufficient light. More importantly, we repeatedly found that the mycorrhizal growth and yield responses of both tested rice varieties were positive or neutral under the non-flooding treatment, while they became negative under flooding conditions, and the mycorrhizal response in the GNZ cultivar, which is supposed to have a relatively high O_2_ supply to the rhizosphere (Wu et al., 2011), was generally more positive than that in the SHZ cultivar. These results strongly suggest that the outcome of AMF symbiosis for rice plants is highly context dependent (see Genre et al., 2020). This study emphasized the high importance of flooding conditions in determining mycorrhizal effects. Gewaily (2019) analyzed the performance of 107 rice cultivars with and without AMF inoculations under field conditions and also found that the beneficial effects of AMF on rice yields reduced with the increase of flooding intensity. These results, together with ours, suggest that the flooding regime is of vital importance in determining the functional role of AMF in rice cultivation systems. The interaction between AMF and rice can be greatly affected by factors such as the identity of AMF species (Chan et al., 2013), rice varieties (Huang et al., 2020), and soil properties (Fiorilli et al., 2013). For example, Solaiman and Hirata (1995) reported that AMF colonization resulted in a decrease in the amount of shoot dry matter under flooded conditions and also in the production of unhulled grains under non-flooded conditions. In another study, they found higher grain yield and P concentration of unhulled grain and shoots upon AMF inoculation in sterilized paddy soil (Solaiman and Hirata, 1996). It must be noted that the present study was conducted in the greenhouse, and only one AMF species and two flooding regimes were applied. This simplified condition is completely different from the complex environment in paddy fields, where rice roots undergo dynamically changing water regimes (Datta et al., 2017), and are co-colonized by different AMF species and many other microbes (Jha and Subramanian, 2014; Chialva et al., 2020). Therefore, the results from this study should be considered with caution. Keeping these limitations in mind, the findings of this study have important implications for not only AMF application in rice production but also our understanding of the AMF functional role in wetland ecosystems.

### C-P Exchange Between Rice and Arbuscular Mycorrhizal Fungi Under Different Flooding and Shading Regimes

The C-P exchange between host plants and AMF is the keystone for AM symbiosis and has been reported to be a key factor in predicting the symbiosis outcome (Johnson, 2010; Smith and Smith, 2011; Corrêa et al., 2014). AMF are completely dependent on a supply of photosynthetically fixed C from their hosts for the growth of intraradical and extraradical structures (Bravo et al., 2016; Jiang et al., 2017; Keymer et al., 2017; Luginbuehl et al., 2017). Previous studies have reported the decrease of C allocation from host plants to AMF under flooding (Tuo et al., 2015; Knegt et al., 2016; Bao et al., 2019). In our study, the significantly decreased AMF colonization and EHD in the flooding environment also implied, to some extent, that rice reduced the C investment to AMF under flooding. There are three possible explanations. First, flooding reduced the C accumulation capacity for rice, as indicated by the decreased photosynthesis efficiency observed in our study. Secondly, although flooding could promote the development of aerenchyma in rice roots, supporting rice adaptation and AMF presence and functionality in wetlands (Vallino et al., 2014; Wang et al., 2015), it may also lead to the decrease of the large lateral roots and cortical cells that are required for AMF development (see Vallino et al., 2014). However, previous works have suggested that the photosynthetic rates of host plants increase upon AMF colonization (Chandrasekaran et al., 2016), possibly due to enhanced rhizospheric sink strength (Gavito et al., 2019). Fiorilli et al. (2018) also identified the up-regulation of genes and proteins involved in photosynthesis and related processes in wheat. In our study, the photosynthetic activity in mycorrhizal rice was lower than that in the non-mycorrhizal plants, especially at the maturing stage. A possible explanation is that AMF colonization promoted the early maturity of rice plants, resulting in the decreased photosynthetic capacity of senescent leaves in mycorrhizal plants. Further studies are needed to identify the mechanisms.

The reduction of mycorrhizal P delivery to rice should partially account for the decrease of MGR under flooding vs. non-flooding conditions, especially considering that the direct P acquisition pathway in mycorrhizal rice plants was suppressed here and in a previous study (Bao et al., 2019). Flooding decreased the mycorrhizal P benefit of rice plants, as indicated by the significant decrease of MPR under flooding conditions in both Exps. 1 and 2. Our previous study also reported a similar decrease of mycorrhizal P benefit in rice under flooding (Bao et al., 2019). The observation that flooding reduced AMF colonization and the density of AMF extraradical hyphae, by which AMF absorb and deliver P to host plants (Smith and Read, 2010), while it did not influence the expression of the mycorrhizal pathway gene *GintPT* (relative to *GintEF2*), implies that the suppression of the mycorrhizal P pathway by flooding was mainly due to a decrease in the number of living AMF hyphae inside and outside roots. Previous studies have also found that flooding reduced the spread of AMF extraradical hyphae, possibly because of O_2_ depletion in flooded soil (Ipsilantis and Sylvia, 2007; Møller et al., 2013). Meanwhile, flooding may also change soil pH and increase the mobility of P in soil (Ding et al., 2019), leading to the decreased reliance of rice plants on the mycorrhizal pathway for P uptake. Furthermore, the C and P exchange is functionally linked in AM symbiosis (Hammer et al., 2011; Kiers et al., 2011), which may explain both the flooding-induced reduction in mycorrhizal P delivery and rice C allocation to AMF. Collectively, the reduction of the mycorrhizal P benefit following flooding in AM symbiosis, observed here and in a previous study (Bao et al., 2019), should be closely related to the flooding-induced inhibitory mycorrhizal responses in rice plants.

The sudden and intensive decrease of light availability to a mycorrhizal plant triggers the rapid deactivation of P transfer from the AMF to the plant (Konvalinková et al., 2015; Konvalinková and Jansa, 2016). In the present study, when we decreased rice C assimilation by shading, mycorrhizal P delivery was reduced, as shown by the reduction of MPR (by 63.7%) and also the shoot P levels (by 11.8%), suggesting the high importance of light supply for the mycorrhizal P effect. Decreased light availability *via* long-term shading reduces AMF biomass, root colonization, and the MGR of plants (Shi et al., 2014; Johnson et al., 2015; Neuenkamp et al., 2021), all of which were also observed under non-flooding conditions in our study, possibly due to the high C cost of the symbiosis relative to the C availability under shading (Fellbaum et al., 2014; Neuenkamp et al., 2021). While under flooding conditions, the shading treatment applied here alleviated the mycorrhizal suppression to rice growth, implying the complex interactions between these environmental factors in impacting the outcome of AM symbiosis. The effects of shading on AM symbiosis are complex and dependent on the intensities and durations of shading (Konvalinková and Jansa, 2016). To achieve a comprehensive understanding of the effects of shading on AM symbiosis, further in-depth study is still needed. Despite this, our results, together with others (Konvalinková and Jansa, 2016; Neuenkamp et al., 2021), highlight the high importance of light supply for the symbiotic functioning of AMF. The positive MGR of plants may eventually become negative when the symbiotic costs (C transferred to the mycorrhiza) outweigh the symbiotic benefits (e.g., P acquisition) [see review in Smith and Smith (2011)]. In the present study, the observation of the inhibitory mycorrhizal responses of rice plants following the shading and/or flooding treatments should be attributed at least in part to the reduction of the mycorrhizal P benefit.

### Mycorrhizal Defense Response of Rice Under Different Flooding and Shading Regimes

In addition to its nutritional effects, AMF symbiosis can also affect the biotic resistance of host plants (Rivero et al., 2018; Campo et al., 2020). Plant basal resistance systems play important roles in the regulation of plant compatibility to AMF (Campos-Soriano et al., 2012; Wang et al., 2021). AMF have evolved the capacity to circumvent defense mechanisms that are controlled by the plant’s immune system (Wang et al., 2021). Still, host plants exhibit some defense responses during their initial contact with AMF (Gutjahr and Paszkowski, 2009; Campos-Soriano et al., 2012), which are switched off after a few days to allow for subsequent colonization in a compatible AMF host (Blilou et al., 1999; Gutjahr and Paszkowski, 2009). This AMF-triggered defense response, namely “defense priming,” can result in a more efficient activation of defense mechanisms in response to attack by potential enemies (Fiorilli et al., 2015, 2018; Gutjahr et al., 2015). In our study, regardless of MGRs, AMF colonization seemed to trigger a constitutive defense response in rice plants cultivated under different flooding and shading environments. This AMF-triggered defense response may enhance the capacity of rice plants to resist disease (Gutjahr et al., 2015; Martinez-Medina et al., 2016). Our findings imply the multifaceted functions of AM symbiosis and their effects on rice performance and suggest that under various environments, AMF application potential should be evaluated from multiple perspectives. Two previous studies have reported the differential mycorrhizal responses among different root types in rice (Fiorilli et al., 2015; Gutjahr et al., 2015), and revealed the AMF-induced defense responses in the fine lateral roots, which are less compatible with AMF colonization. In our study, the whole rice lateral root system was used for molecular analysis, and the observation of the AMF-induced constitutive defense response may represent the incompatible reaction from the fine lateral roots. Interestingly, the shading-induced carbon starvation in rice plants significantly increased the strength of the AMF-triggered defense responses in this work. A similar result has also been reported in Neuenkamp et al. (2021), who found that low light availability can reduce the compatibility of plant roots to AMF colonization. The strengthened defense response to the fungal partner could partially explain the reduced AMF colonization and MPR in shading conditions, as observed here and in other studies (Konvalinková et al., 2015; Konvalinková and Jansa, 2016). These results imply, to some extent, a fine-tuning of AM symbiosis in rice plants according to ever-changing environments. In other words, according to the richness of carbon sources, rice roots finely coordinate their defense responses to modulate the carbon-consuming AMF (Neuenkamp et al., 2021). More in-depth study is still needed to explore this mechanism.

## Conclusion

**FIGURE 6:**
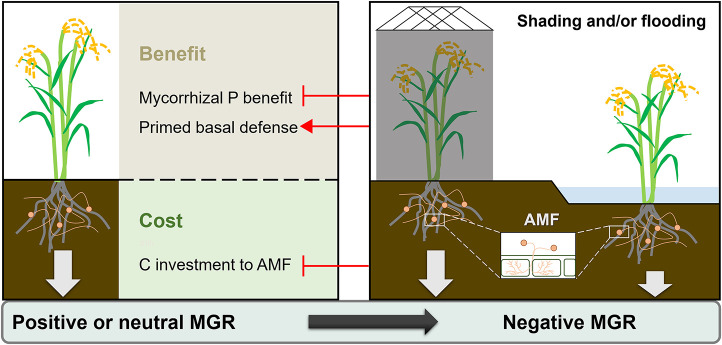
Proposed model showing the effects of flooding or/and shading on the mycorrhizal growth response (MGR) of rice plants. ⊣: inhibitory effect, →: promotive effect.

This study found that flooding and shading both modulated the outcome of AMF symbiosis for rice plants (Figure 6). The positive or neutral mycorrhizal growth and yield responses of rice in non-flooding environments became negative under flooding and/or shading conditions, which should be attributed, at least in part, to the reduction of mycorrhizal P benefit in these environments. Regardless of MGRs, rice roots showed a constitutive defense response upon AMF colonization, especially in shading conditions, implying the multifaceted functions of AMF symbiosis on rice performances. These results have important implications for the application of AMF in rice-production systems. Further in-depth studies are needed to identify the mechanisms involved in the flooding-induced inhibitory mycorrhizal effect, as well as in the modulation of mycorrhizal association in rice plants.

## Data Availability Statement

The original contributions presented in the study are included in the article/Supplementary Material, further inquiries can be directed to the corresponding author.

## Author Contributions

YW proposed the study and designed the experiments. XB and YW conducted the experiments and wrote the manuscript. YW and SL supervised the study. All authors approved the submitted manuscript.

## Conflict of Interest

The authors declare that the research was conducted in the absence of any commercial or financial relationships that could be construed as a potential conflict of interest.

## Publisher’s Note

All claims expressed in this article are solely those of the authors and do not necessarily represent those of their affiliated organizations, or those of the publisher, the editors and the reviewers. Any product that may be evaluated in this article, or claim that may be made by its manufacturer, is not guaranteed or endorsed by the publisher.

## Footnotes

1
www.mycagrolab.com
