# Supplementary material for: Effects of Arbuscular Mycorrhizal Fungi on Rice Growth Under Different Flooding and Shading Regimes
Source: Front Microbiol. 2021 Oct 26;12:756752. doi: 10.3389/fmicb.2021.756752 (PMC8577809; doi:10.3389/fmicb.2021.756752)
Supplement: Supplementary file 2 [file Data_Sheet_2.docx]

**Supplementary** **Materials**

**Title:** Effects of arbuscular mycorrhizal fungi on rice growth under different flooding and shading regimes

**Authors:** Yutao Wang ^†1^, Xiaozhe Bao ^†^^[[1]](#footnote-2)^, Shaoshan Li*

**Correspondence:** [lishsh@scnu.edu.cn](mailto:lishsh@scnu.edu.cn)

**The Supplementary Materials file includes**

Supplementary Figures 1–6

Supplementary Tables 1 and 2


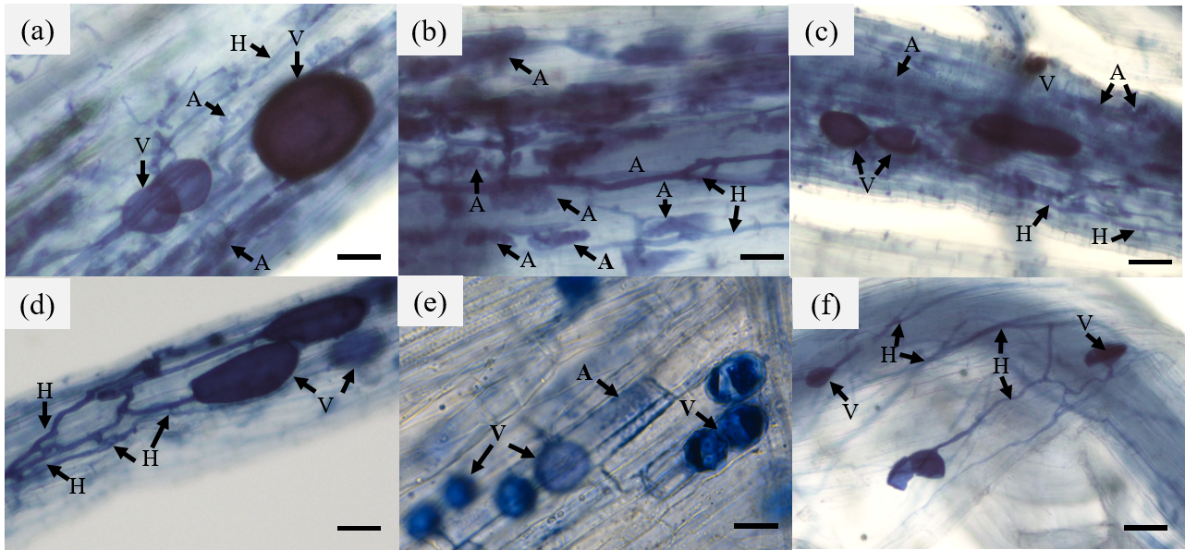


**Supplementary Figure 1.** Typical arbuscular (A), vesicle (V), and hyphal (H) structures observed in rice roots under non-flooding (a, b), intermittent flooding (c, d), and continuous flooding (e, f) treatments; bar length: 20 μm.

**
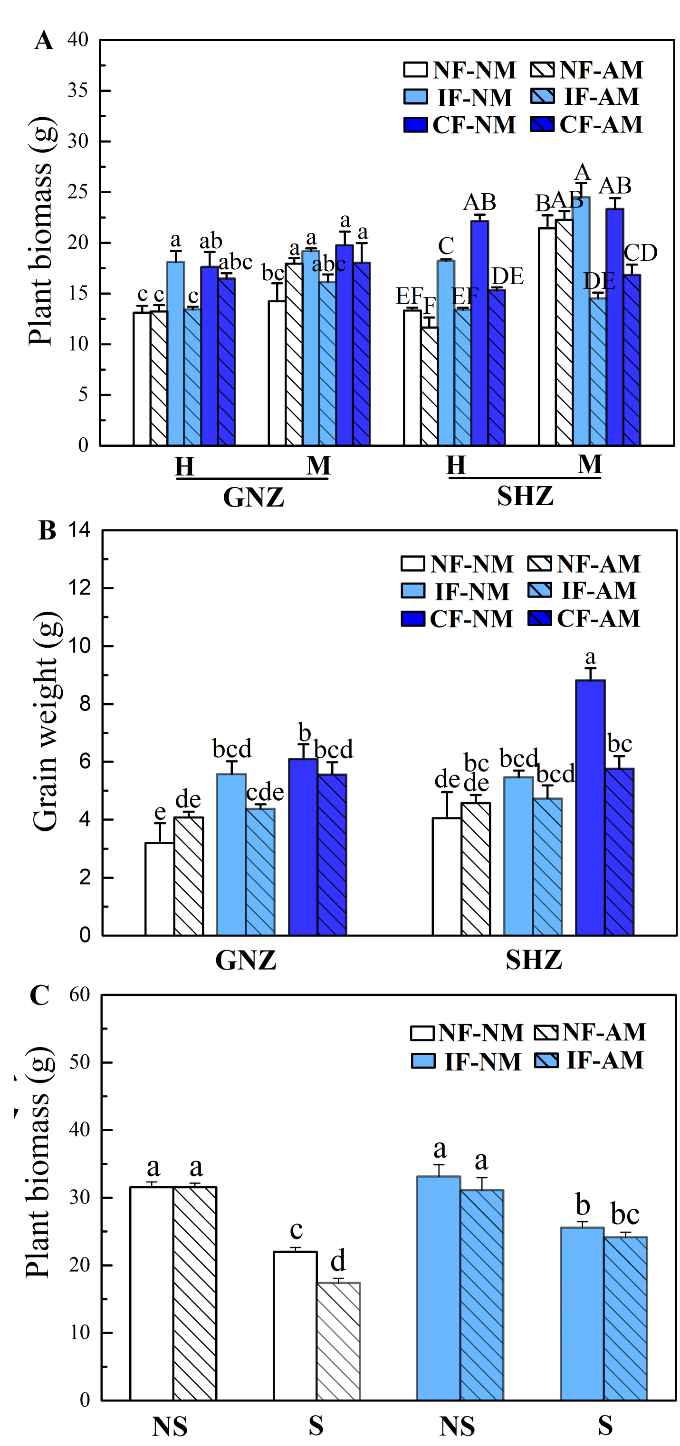
**

**Supplementary Figure 2.** The plant biomass and grain weight of mycorrhizal (AM) and non-mycorrhizal (NM) rice plants under different flooding (NF: non-flooding, IF: intermittent flooding, CF: continuous flooding) and shading (S: shading, NS: non-shading) treatments in Exp. 1 (A, B) and Exp. 2 (C). In Exp. 1, the rice varieties Guinongzhan (GNZ) and Sanhuangzhan (SHZ) were harvested at the heading (H) and maturing (M) stages; in Exp. 2, the rice variety GNZ was harvested at the heading stage. Different letters above the columns indicate significant differences at the *P*<0.05 level (In Fig. S1A, lowercase and uppercase letters are used for GNZ and SHZ cultivar, respectively). Means and standard errors from five replicates are shown.


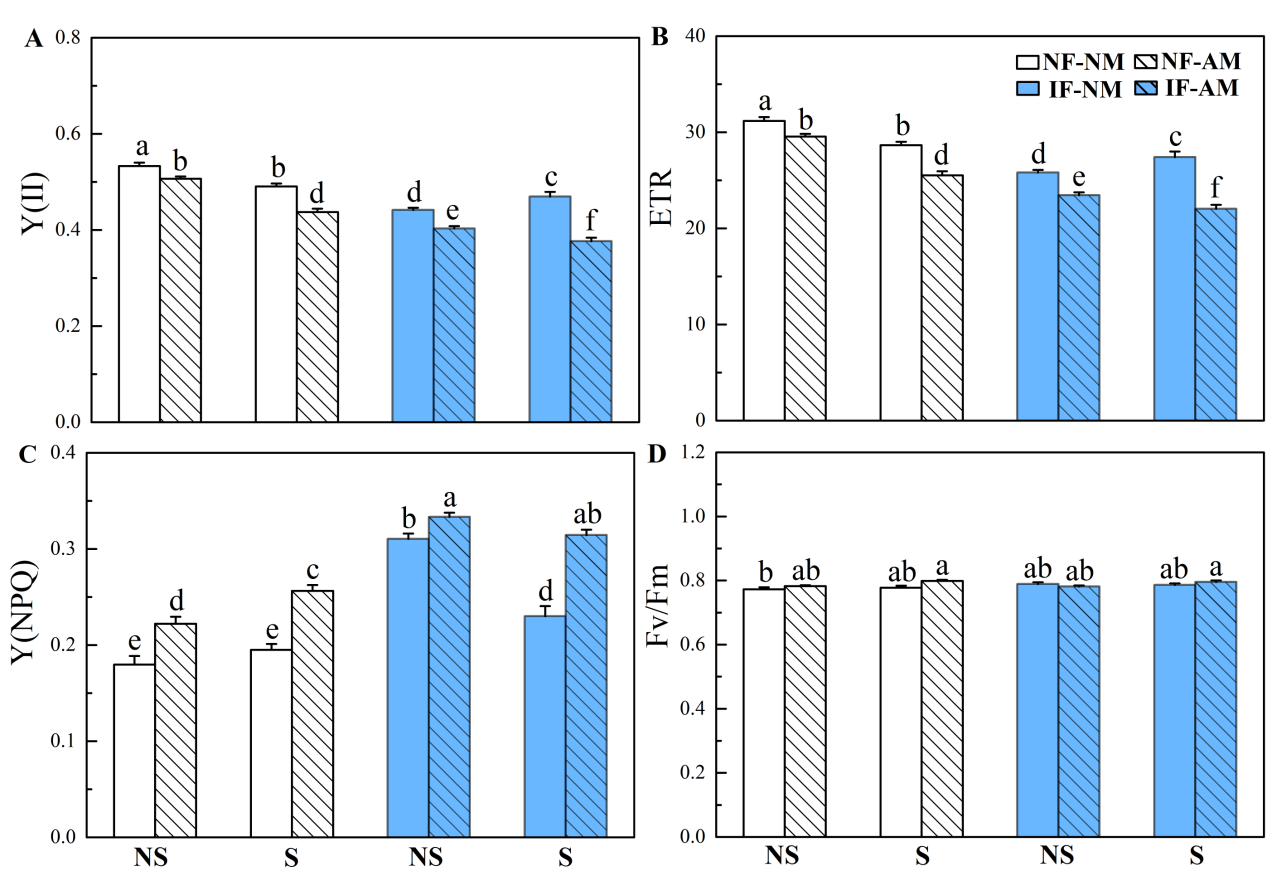


**Supplementary Figure 3.** Rice chlorophyll II fluorescence parameters under different flooding (IF: intermittent flooding, NS: non-flooding) and shading (S: shading, NS: non-shading) conditions in Exp. 2. (A) Maximum quantum yield (Fv/Fm), (B) actual quantum yield (Y(II)), (C) the electron transport rate (ETR), and (D) the yield of non-photochemical quenching (Y(NPQ)) in the mycorrhizal (AM) and non-mycorrhizal (NM) leaves of Guinongzhan (GNZ) collected at the heading stage. Different letters above the columns indicate significant differences at the *P*<0.05 level. Means and standard errors from five replicates are shown.

**
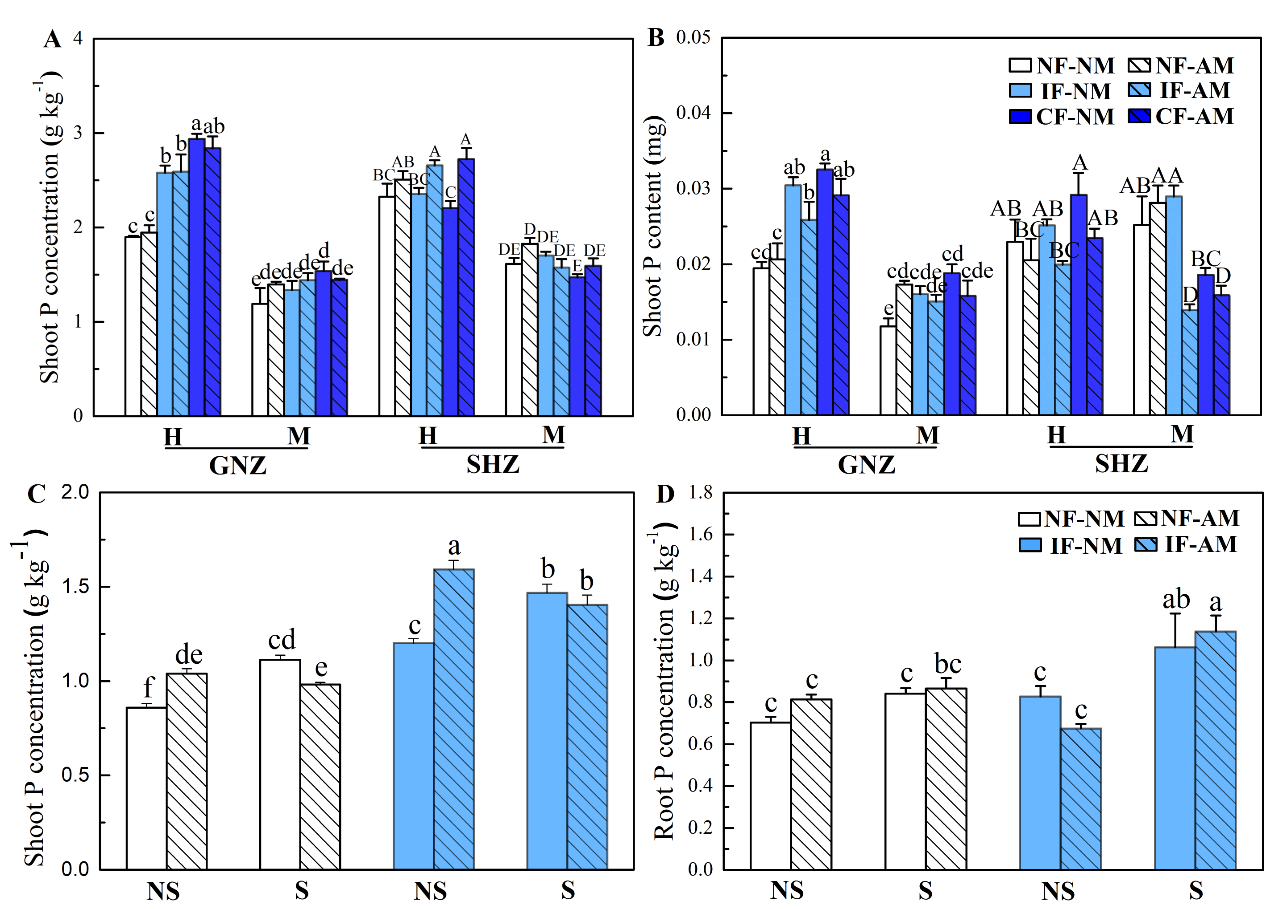
**

**Supplementary Figure 4.** Plant P status in mycorrhizal (AM) and non-mycorrhizal (NM) rice plants under different flooding (NF: non-flooding, IF: intermittent flooding, CF: continuous flooding) and shading (S: shading, NS: non-shading) treatments in Exp. 1 (A, B) and Exp. 2 (C, D). In Exp. 1, the rice varieties Guinongzhan (GNZ) and Sanhuangzhan (SHZ) were harvested at the heading (H) and maturing (M) stages; in Exp. 2, the rice variety GNZ was harvested at the heading stage. Different letters above the columns indicate significant differences at the *P*<0.05 level (In Fig. S3A and B, lowercase and uppercase letters are used for GNZ and SHZ, respectively). Means and standard errors from three (A, B) and five (C, D) replicates are shown.

**
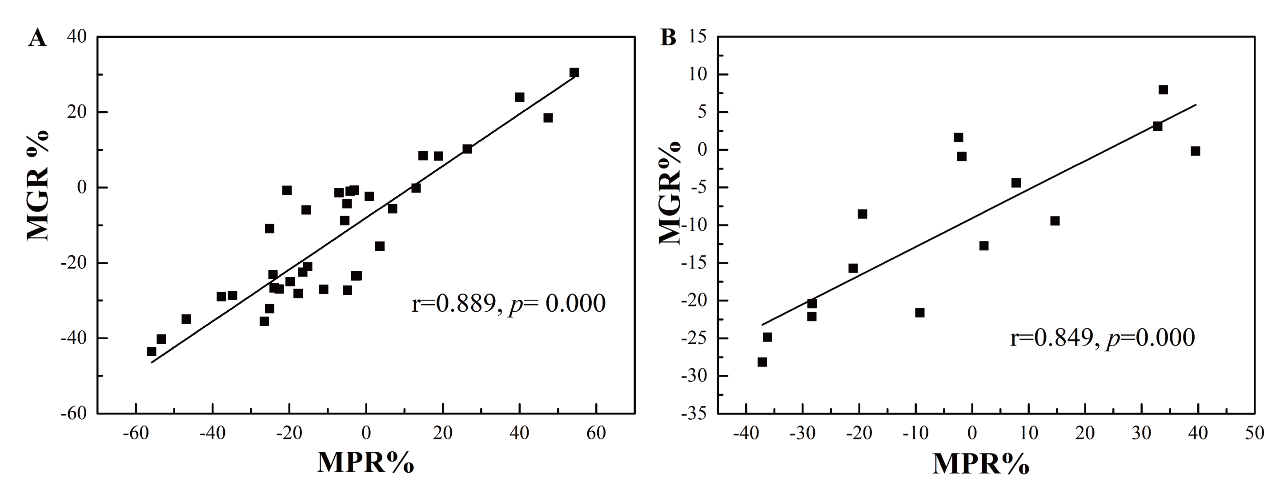
**

**Supplementary Figure 5.** Correlation between mycorrhizal growth response (MGR) and mycorrhizal phosphorus response (MPR) in Exp. 1 (A) and Exp. 2 (B). Means from three (A) and five replicates (B) are shown.


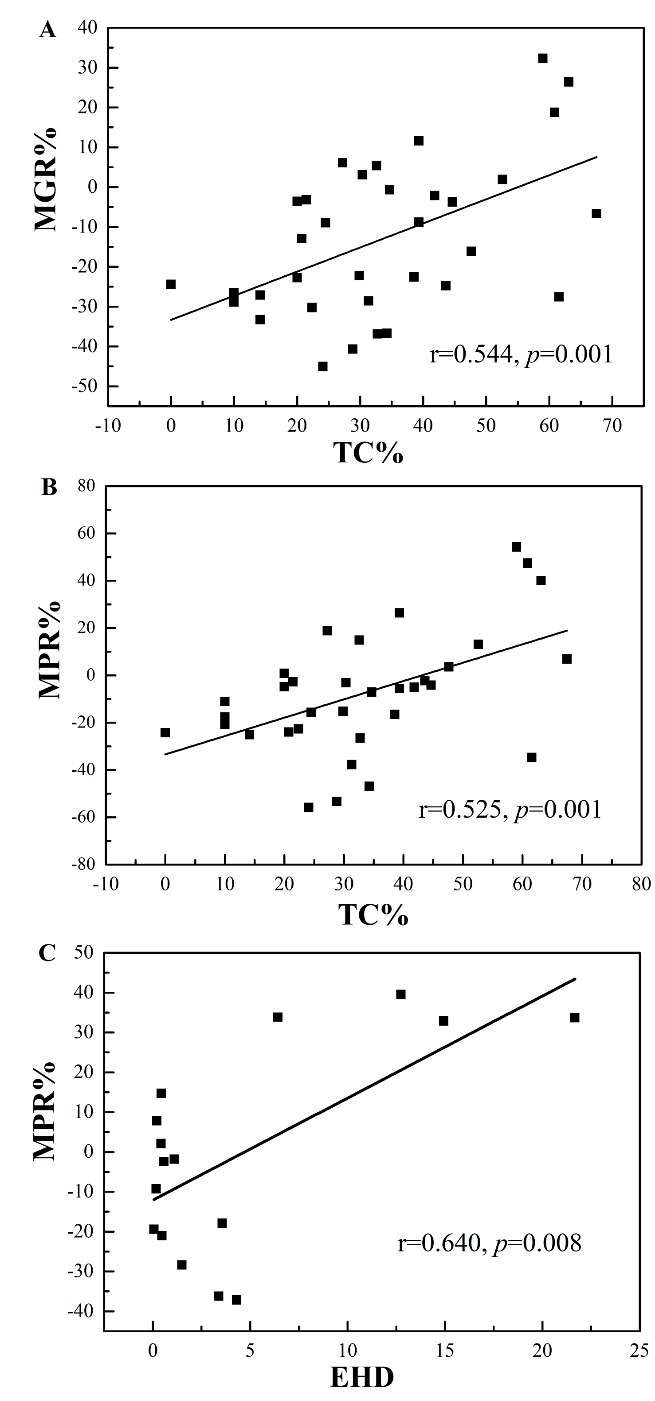


**Supplementary Figure 6.** Correlation between mycorrhizal growth response (MGR) and mycorrhizal P response (MPR) with the total colonization rates of arbuscular mycorrhizal fungi (TC%) in Exp. 1 (A, B), and the correlation between MPR and extraradical hyphal density (EHD) in Exp. 2 (C). Means from three (A, B) and five (C) replicates are shown. **Supplementary Table 1.** Primer sequences of the genes involved in the direct P pathway, mycorrhizal P pathway, and common defense system in rice roots

| **Gene name** | **Primer sequences (5’ to 3’)** |  |
| --- | --- | --- |
|  |  |  |
| *Oscyclophilin2* | Forward: GTGGTGTTAGTCTTTTTATGAGTTCGT |  |
|  | Reverse: ACCAAACCATGGGCGATCT |  |
| *GintEF1α* | Forward: GCTATTTTGATCATTGCCGCC | |
|  | Reverse: TCATTAAAACGTTCTTCCGACC | |
| *OsPT2* | Forward: GACGAGACCGCCCAAGAAG |  |
|  | Reverse: TTTTCAGTCACTCACGTCGAGAC |  |
| *OsPT6* | Forward: CCGCCCCTGCAAACTGTA |  |
|  | Reverse: CAACTGGCGGTTTCTTCGAT |  |
| *OsPT11* | Forward: GAGAAGTTCCCTGCTTCAAGCA |  |
|  | Reverse: GAGAAGTTCCCTGCTTCAAGCA |  |
| *OsPR1* | Forward: GGAGAAGGGCTCCTACGACT |  |
|  | Reverse: GGGGAAGTAGGTGCAGATGA |  |
| *OsPBZ1* | Forward: GCGATGGCTCCTGTGTGG |  |
|  | Reverse: CTCCGGCGACAGTGAGCT |  |
| *GintPT* | Forward: AACACGATGTCAACAAAGCAAC |  |
|  | Reverse: AAGACCGATTCCATAAAAAGCA |  |

**Supplementary Table 2.** Analysis of parameter estimates from generalized linear models in Exp. 1: effects of arbuscular mycorrhizal fungi (AMF) status, rice cultivars, rice growth stage, and flooding treatments on tested plants and AMF parameters.

| **Variables** | **AM status** | |  | **Cultivars** | |  | **Stage** | |  | **Flooding** | |
| --- | --- | --- | --- | --- | --- | --- | --- | --- | --- | --- | --- |
|  | **F** | **Sig.** |  | **F** | **Sig.** |  | **F** | **Sig.** |  | **F** | **Sig.** |
| **Growth parameters** |  |  |  |  |  |  |  |  |  |  |  |
| Plant biomass | 55.2 | *** |  | 16.7 | *** |  | 76.3 | *** |  | 15.9 | *** |
| Grain weight | 6.38 | * |  | 7.68 | * |  | -- | -- |  | 30.7 | *** |
| Mycorrhizal growth response | -- | -- |  | 40.6 | *** |  | 5.28 | * |  | 47.6 | *** |
| Mycorrhizal yield response | -- | -- |  | 4.51 | ns |  | -- | -- |  | 27.3 | *** |
| **Plant C assimilation** |  |  |  |  |  |  |  |  |  |  |  |
| Fv/Fm | 3.38 | ns |  | 16.5 | *** |  | 59.7 | *** |  | 4.01 | * |
| Y(II) | 93.2 | *** |  | 1.15 | ns |  | 676 | *** |  | 283 | *** |
| ETR | 93.2 | *** |  | 1.15 | ns |  | 676 | *** |  | 283 | *** |
| Y(NPQ) | 60.2 | *** |  | 26.9 | *** |  | 333 | *** |  | 331 | *** |
| **AMF structures** |  |  |  |  |  |  |  |  |  |  |  |
| VC% | -- | -- |  | 1.01 | ns |  | 14.7 | *** |  | 31.3 | *** |
| HC% | -- | -- |  | 9.07 | ** |  | 9.03 | ** |  | 44.4 | *** |
| AC% | -- | -- |  | 4.53 | * |  | 74.5 | *** |  | 34.1 | *** |
| TC% | -- | -- |  | 0.63 | ns |  | 72.1 | *** |  | 63.4 | *** |
| **AMF P delivery to plants** |  |  |  |  |  |  |  |  |  |  |  |
| Mycorrhizal P response | -- | -- |  | 17.5 | *** |  | 2.26 | ns |  | 25.3 | *** |
| Shoot P conc. | 9.38 | ** |  | 9.81 | ** |  | 641 | *** |  | 16.5 | *** |
| Shoot P content | 14.1 | *** |  | 4.60 | * |  | 68.6 | *** |  | 2.81 | ns |

**Note:** The chlorophyll fluorescence parameters (Fv/Fm, Y(II), ETR, and Y(NPQ)) are indicative of plant C assimilation; Fv/Fm: maximum quantum yield; Y(II): actual quantum yield; ETR: electron transport rate, Y(NPQ): yield of non-photochemical quenching. VC%, HC%, AC%, and TC% indicate vesical, hyphal, arbuscular, and total colonization intensities, respectively; --, not applicable; ns, not significant at the 0.05 probability level; *, **, and *** represent statistical significance at the 0.05, 0.01, and 0.001 probability levels, respectively.

1. [↑](#footnote-ref-2)
